# MOMAST^®^ Reduces the Plasmatic Lipid Profile and Oxidative Stress and Regulates Cholesterol Metabolism in a Hypercholesterolemic Mouse Model: The *Proof of Concept* of a Sustainable and Innovative Antioxidant and Hypocholesterolemic Ingredient

**DOI:** 10.3390/antiox12071335

**Published:** 2023-06-24

**Authors:** Ivan Cruz-Chamorro, Guillermo Santos-Sánchez, Eduardo Ponce-España, Carlotta Bollati, Lorenza d’Adduzio, Martina Bartolomei, Jianqiang Li, Antonio Carrillo-Vico, Carmen Lammi

**Affiliations:** 1 Instituto de Biomedicina de Sevilla (IBiS), Hospital Universitario Virgen del Rocío, Consejo Superior de Investigaciones Científicas (CSIC), Universidad de Sevilla, 41013 Seville, Spain; icruz-ibis@us.es (I.C.-C.); gsantos-ibis@us.es (G.S.-S.); vico@us.es (A.C.-V.); 2 Departamento de Bioquímica Médica y Biología Molecular e Inmunología, Facultad de Medicina, Universidad de Sevilla, 41009 Seville, Spain; 3 Department of Pharmaceutical Sciences, University of Milan, 20133 Milan, Italy; carlotta.bollati@unimi.it (C.B.); martina.bartolomei@unimi.it (M.B.);

**Keywords:** LDL, MOMAST^®^, Monacolin K, red yeast rice, PCSK9

## Abstract

MOMAST^®^ is a patented natural phenolic complex, rich in tyrosol (9.0 g/kg, Tyr), hydroxityrosol (43,5 g/kg, OH-Tyr), and verbascoside (5.0 g/Kg), which is obtained from the OVW by-product of the *Coratina* cultivar with potent direct antioxidant activity (measured by DPPH and FRAP assays, respectively). Indeed, MOMAST^®^ represents an innovative sustainable bioactive ingredient which has been obtained with ethical and empowering behavior by applying the principles of a circular economy. In the framework of research aimed at fostering its health-promoting activity, in this study it was clearly demonstrated that MOMAST^®^ treatment reduced the oxidative stress and levels of total cholesterol (TC) and low-density lipoprotein (LDL) cholesterol, and increased the HDL levels, without changes in the triglyceride (TG) levels in Western diet (WD)-fed mice. The modulation of the plasmatic lipid profile is similar to red yeast rice (RYR) containing Monacolin K (3%). In addition, at the molecular level in liver homogenates, similarly to RYR, MOMAST^®^ exerts cholesterol-lowering activity through the activation of LDL receptor, whereas, unlike RYR, MOMAST^®^ reduces proprotein convertase subtilisin/kexin type 9 (PCSK9) protein levels via hepatic nuclear factor 1 (HNF1)-α activation. Hence, this study provides the *proof of concept* regarding the hypocholesterolemic activity of MOMAST, which could be successfully exploited as an active ingredient for the development of innovative and sustainable dietary supplements and functional foods.

## 1. Introduction

During the olive oil extraction process, only a small percentage of polyphenols of the total present in the olives is transferred to the oil, while an important percentage is retained in the olive oil vegetation water (OVW) [1,2]. The quantity of phenols is very variable in relation to the type of olives, their state of ripeness, storage, and the degradation that they had to undergo between harvesting and processing [3]. In this context, MOMAST^®^ is a patented natural phenolic complex, rich in tyrosol (9.0 g/kg, Tyr) hydroxityrosol (43.5 g/kg, OH-Tyr), and verbascoside (5.0 g/kg), which is obtained from the OVW by-product of the *Coratina* cultivar using exclusively physical and mechanical methods, without the use of solvents and other chemical processes [4]. The high content of both Tyr and OH-Tyr is clearly due to the hydrophilic nature of these phenolic compounds which tend to accumulate in the OVW. In light of these observations, MOMAST^®^ represents an innovative sustainable bioactive ingredient which has been obtained through ethical and empowering behavior by applying the principles of a circular economy which are perfectly in line with the sustainable development goals (SDG) of the 2030 Agenda and the Farm to Fork Strategy [5].

The literature widely reports the antioxidant properties of polyphenols both at the cellular and in vivo levels [6,7]. In agreement, given that its composition is rich in Tyr and OH-Tyr, MOMAST^®^ exerts both antioxidant and anti-inflammatory activities in isolated rat tissues after LPS stimulus [8]. Interestingly, MOMAST^®^ displays a hypocholesterolemic property through a mechanism of action which is not only correlated to the antioxidant or anti-inflammatory effect, but to its direct ability to modulate the activity of 3-hydroxy-3-methylglutaryl Coenzyme A reductase (HMGCoAR), the enzyme targeted by statins or by Monacolin K, which is present in red yeast rice (RYR) [4]. In more detail, MOMAST^®^ reduced the in vitro enzyme activities with a dose–response trend and an IC_50_ equal to 75 µg/mL. Through the HMGCoAR inhibition, MOMAST^®^ activated the intracellular cholesterol pathway with a dual mechanism of action, leading to an increase in low-density lipoprotein (LDL) receptor (LDLR) and HMGCoAR protein levels via sterol regulatory element binding protein (SREBP)-2 activation. In this context, dedicated experiments confirmed that MOMAST^®^ specifically increases the LDLRs localized on the surface of human hepatic HepG2 cells. In addition, unlike statin, MOMAST^®^ significantly reduced the hepatic nuclear factor 1 (HNF1)-α, leading to a decrease in mature proprotein convertase subtilisin/kexin type 9 (PCSK9) levels and to a functional reduction in its secretion in extracellular environments [4]. In agreement with evidence from the literature [9,10], the reduction in PCSK9 production and secretion along with the selective improvement of LDLRs localized on the surface of hepatocytes led, from a functional point of view, to an increased ability of hepatic cells to absorb LDL from the extracellular environment with a final in vitro hypocholesterolemic effect [4].

In light of the evidence obtained at the in vitro level [4,8], the present study aimed at obtaining the *proof of concept* of the antioxidant and hypocholesterolemic activities of MOMAST^®^ by performing a dedicated in vivo study. Notably, the direct antioxidant activity of MOMAST^®^ was determined by performing DPPH and FRAP assays, respectively. Furthermore, an in vivo study on C57BL/6 mice fed a Western diet (WD) was carried out to evaluate the antioxidant activity of MOMAST^®^ at the plasmatic level and its effect on the plasmatic lipid profile (measuring total cholesterol (TC), LDL and high-density lipoprotein (HDL), and triglyceride (TG) parameters) and the liver cholesterol metabolism pathway.

## 2. Materials and Methods

### 2.1. Chemicals

All chemicals and reagents were commercially available, and more details are reported in the Supplementary Materials.

#### MOMAST^®^ Description

Bioenutra S.R.L. (Ginosa (TA) Italy) supplied the patented MOMAST^®^ sample directly from the production process. MOMAST^®^ sample is a phenolic complex, rich in tyrosol (9.0 g/kg, Tyr), hydroxityrosol (43.5 g/kg, OH-Tyr), and verbascoside (5.0 g/Kg). Its chromatogram is available as a Supplementary Material (Figure S1).

### 2.2. In Vitro MOMAST^®^ Antioxidant Activity

#### 2.2.1. 2,2-Diphenyl-1-picrylhydrazyl (DPPH) Assay

To estimate the radical scavenging capacity of MOMAST^®^, the DPPH assay was carried out. More details are reported in the Supplementary Materials.

#### 2.2.2. Ferric Reducing Antioxidant Power (FRAP) Assay

A volume of 15 μL of a sample containing MOMAST^®^ was combined with 140 μL of FRAP solution, which consisted of 0.83 mM TPTZ, 1.66 mM FeCl_3_× 6H_2_O, and 0.25 M acetate buffer (pH 3.6). More experimental details are provided in Supplementary Materials.

### 2.3. Study Design

**Figure 1:**
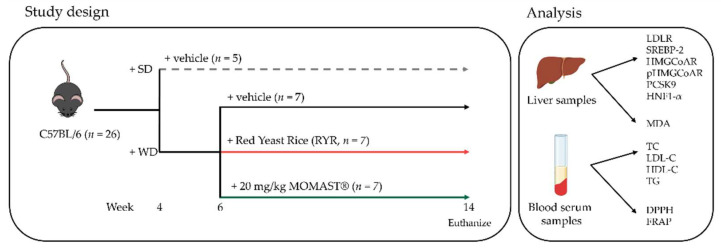
Schematic representation of the study design, and of lipidic and antioxidant parameters analysis. SD, standard diet; WD, Western diet.

Twenty-six C57BL/6 4-week-old mice were housed at the Animal Facility of the Instituto de Biomedicina de Sevilla (IBiS) under standard conditions and were fed with a WD (45% energy from fat, TestDiet, St. Louis, MO, USA, *n* = 21) or with a standard diet (SD, rodent maintenance diet, ENVIGO, IN, USA, *n* = 5). When mice were 6 weeks old, they were separated into three groups and intragastrically treated with (1) saline (control group, C, *n* = 7), (2) red yeast rice (3% Monacolin K, RYR, ADVA s.r.l., Milan, Italy, *n* = 7), or (3) MOMAST^®^ (Bioenutra s.r.l., Ginosa (TA) Italy, *n* = 7) at 20 mg/kg for eight weeks (Figure 1). Further details are available in the Supplementary Materials.

### 2.4. Biochemical Parameters

All biochemical parameters were quantified in the serum samples using the COBAS E601 modular analyzer (Roche Diagnostic, Basel, Switzerland). Castelli risk indexes (CRI) I and II were calculated as TC/HDL and LDL/HDL, respectively.

### 2.5. In Vivo Antioxidant Activity of MOMAST^®^

#### 2.5.1. DPPH and FRAP Assays

Serum antioxidant capacity was evaluated using DPPH and FRAP assays and both tests were carried out according to the manufacturer’s instructions. See Supplementary Materials for further details.

#### 2.5.2. Determination of Hepatic Malondialdehyde (MDA) Levels

Liver samples were processed following the manufacturer’s instructions. Experimental details are reported in the Supplementary Materials.

### 2.6. Western Blot Analysis

Tissues were homogenized in a lysis buffer and the protein concentration was determined by Bradford’s method. Western Blot experiments were performed using primary antibodies against SREBP-2, HMGCoAR, LDLR, phospho HMGCoAR (Ser872), PCSK9, HNF1-α, and β-actin following conditions previously reported. See Supplementary Materials for further details.

### 2.7. Statistical Analysis

All the data sets were checked for normal distribution by D’Agostino and Pearson test. Since they are all normally distributed with *p*-values < 0.05, statistical analysis was carried out by one-way ANOVA followed by Tukey’s post hoc analysis (GraphPad Software 9, San Diego, CA, USA). Values were expressed as means ± standard deviation; *p*-values ≤ 0.05 were considered to be significant.

## 3. Results

### 3.1. MOMAST^®^ Has In Vitro DPPH Radical Scavenging and Ferric Reducing Capacities

**Figure 2:**
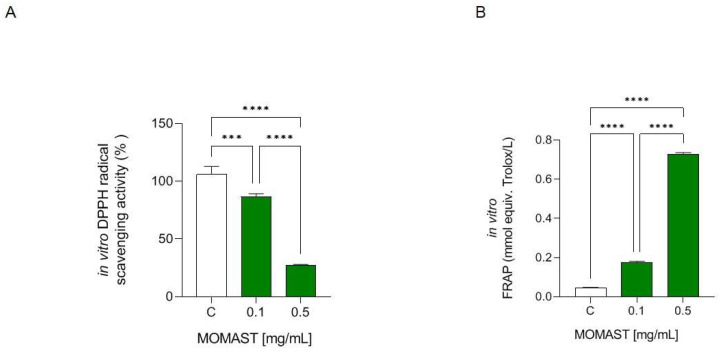
In vitro DPPH radical scavenging (**A**) and ferric reducing capacities (**B**) of MOMAST^®^. Data are represented as mean ± standard deviation. ***, *p* ≤ 0.001; ****, *p* ≤ 0.0001; C: control; ns: no statistical differences.

As Figure 2A shows, MOMAST^®^ reduced the DPPH radical by 13.33 ± 2.858% and 72.57 ± 0.61% at. 0.1 and 0.5 mg/mL, respectively. Regarding the effect of MOMAST^®^ on the capacity to reduce Fe^3+^ to Fe^2+^, it is able to improve the FRAP levels tested at 0.1 mg/mL (0.175 mmol equiv. Trolox/L) and 0.5 mg/mL (0.728 mmol equiv. Trolox/L), versus the control (H_2_O) (Figure 2B).

### 3.2. Effect of MOMAST^®^ on Body Weight

**Figure 3:**
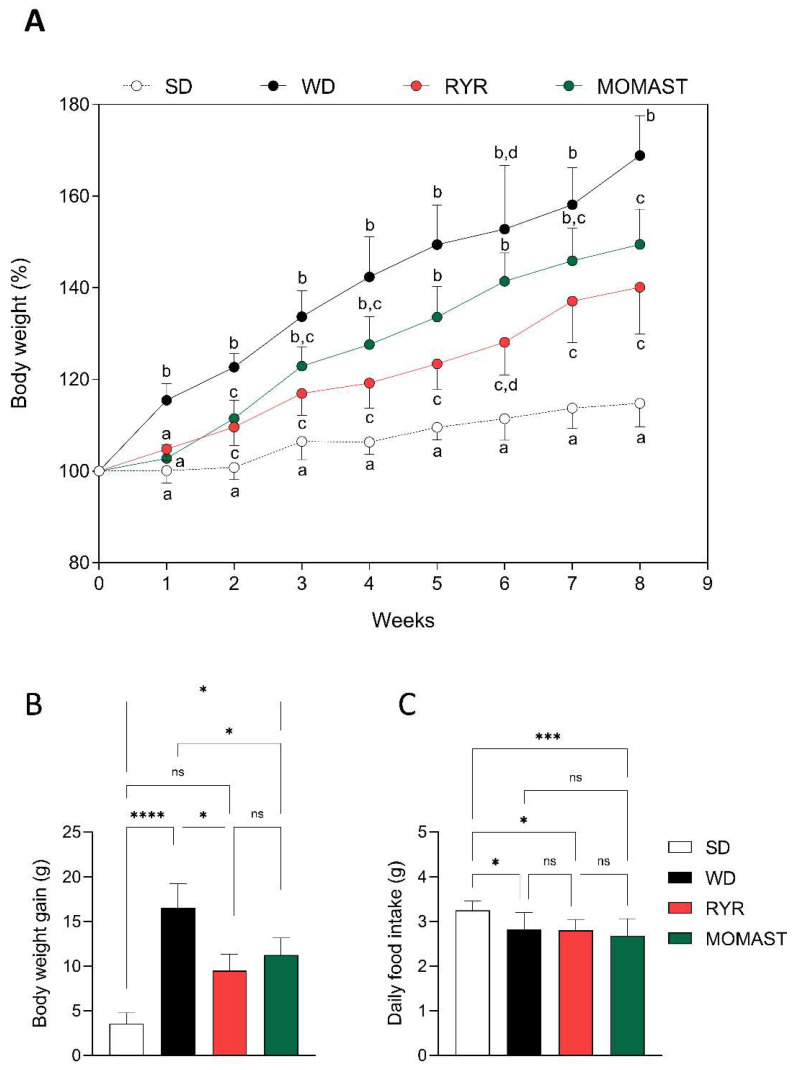
Effects of the MOMAST^®^ on body weight. Body weight monitored over time on each group (**A**), body weight gain (**B**), daily food intake (**C**). Data are represented as mean ± standard deviation. Different letters represent a statistical difference (*p* ≤ 0.05). *, *p* ≤ 0.05; ***, *p* ≤ 0.001; ****, *p* ≤ 0.0001; ns, no statistical differences.

As shown in Figure 3A, WD-fed mice showed a higher increase in body weight throughout the experiment in comparison to the SD-fed mice. This effect was palliated by the treatment with MOMAST^®^ and RYR, whose body weight increase was significantly lower than the WD group. This fact was reflected in the body weight gain (Figure 3B), which was significantly lower in the groups treated with MOMAST^®^ (11.25 ± 1.93 g, *p* = 0.036) and RYR (9.5 ± 1.83 g, *p* = 0.011) than in the WD group (16.57 ± 2.66 g). Regarding the daily food intake (Figure 2C), WD-fed mice showed a lower food ingestion (2.83 ± 0.38 g/mouse/day) in comparison to the SD-fed mice (3.25 ± 0.21 g/mouse/day) (*p* = 0.023). However, no significant differences were found between the WD group and the groups treated with MOMAST^®^ (2.69 ± 0.38 g/mouse/day, *p* = 0.55) and RYR (2.80 ± 0.25 g/mouse/day, *p* = 0.99).

### 3.3. MOMAST^®^ Exerts Antioxidant Effects at Serum and Hepatic Levels

**Figure 4:**
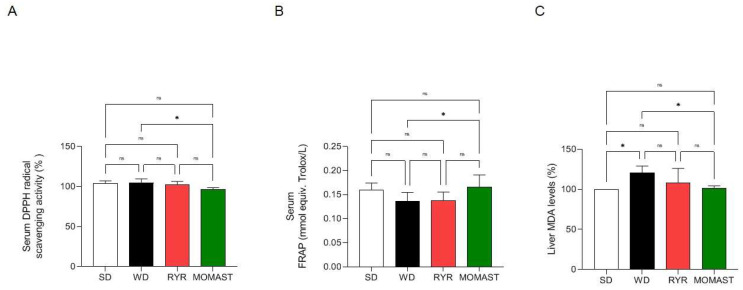
(**A**) DPPH radical scavenging and (**B**) ferric reducing capacities of MOMAST^®^ in serum. (**C**) Modulation of lipid peroxidation in liver. Data are represented as mean ± standard deviation. *, *p* ≤ 0.05; ns: no statistical differences.

Regarding the antioxidant effect on serum, as indicated Figure 4A, MOMAST^®^-fed mice showed an ability to decrease the DPPH radical by 3.51 ± 2.11% compared with WD-fed mice, which showed an increase in DPPH radical of 4.60 ± 4.80% at the serum level. As indicated in Figure 4B, MOMAST^®^-treated mice showed an increase in the FRAP levels (0.17 mmol equiv. Trolox/L) compared to the WD-fed mice (0.14 mmol equiv. Trolox/L, *p* = 0.039). There were no significant differences between the SD and WD groups (*p* = 0.43), nor between the WD and RYR groups (*p* = 0.99).

Moreover, the capacity of MOMAST^®^ to regulate the lipid peroxidation in mice livers was assessed by the MDA evaluation (Figure 4C). As illustrated in Figure 4C, WD-fed mice showed an increase in MDA levels in comparison to the SD-fed mice. In fact, MDA levels were augmented by 121.0 ± 8.12 % in WD-fed mice, whereas the treatment with MOMAST^®^ resulted in a reduction in MDA levels to 101.2 ± 3.33%, restoring the SD lipid peroxidation baseline levels. On the contrary, no significant decrease was observed in the RYR group, in which the MDA levels were increased by 108.1 ± 18.04 vs. the SD group.

### 3.4. MOMAST^®^ Improves the Plasmatic Lipidic Profile

**Figure 5:**
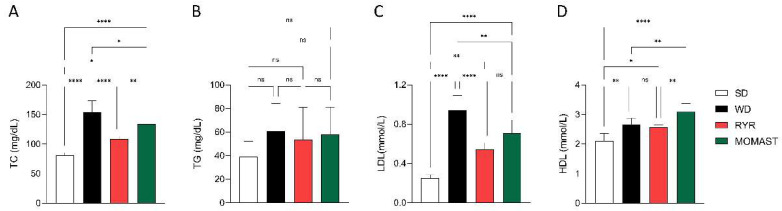
Effects of the MOMAST^®^ on plasma lipidic profile. Plasma concentration of total cholesterol (**A**), triglycerides (**B**), low-density lipoprotein (**C**), and high-density lipoprotein (**D**). Data are represented as mean ± standard deviation. *, *p* ≤ 0.05; **, *p* ≤ 0.01; ****, *p* ≤ 0.0001; ns, no statistical differences.

To investigate the in vivo lipid-lowering effect of MOMAST^®^, the plasma lipid profile and the main cardiovascular risk indexes were analyzed. As shown in Figure 5, WD ingestion increased the TC (153.60 ± 19.65 mg/dL, *p* < 0.0001), LDL (0.94 ± 0.15 mmol/L, *p* < 0.0001), and HDL (2.66 ± 0.22 mmol/L, *p* = 0.004) values, in comparison to the SD-fed mice (TC: 81.32 ± 3.86 mg/dL; LDL: 0.25 ± 0.03 mmol/L; HDL: 2.10 ± 0.26 mmol/L). The treatment with MOMAST^®^ for 8 weeks palliates these effects. Specifically, the treatment reduced by 12.5% (134.40 ± 12.86 mg/dL, *p* = 0.017) and 24.47% (0.71 ± 0.13 mmol/L, *p* = 0.002) the levels of TC and LDL, respectively. In addition, MOMAST^®^ treatment increased the levels of HDL by 16.16% (3.09 ± 0.29 mmol/L, *p* = 0.004). Interestingly, the values in the MOMAST^®^-treated mice were similar to those treated with RYR. More in detail, RYR treatment showed lower TC (108.30 ± 4.96 mg/dL, *p* < 0.0001) and LDL (0.54 ± 0.07 mmol/L, *p* < 0.0001) levels, in comparison to the WD group, without differences in HDL values (*p* = 0.92). No significant differences between groups were found in TG levels (*p* > 0.05).

**Figure 6:**
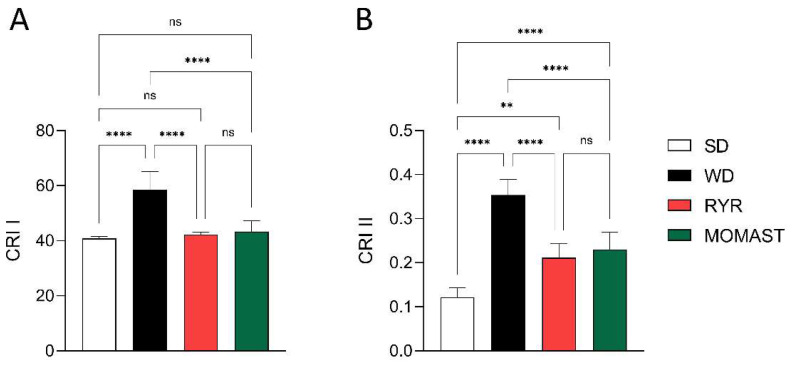
Assessment of the effects of MOMAST^®^ on cardiovascular disease risk indexes. Evaluation of cardiovascular disease risk through Castelli risk index (CRI) I (TC/HDL) (**A**) and II (LDL/HDL) (**B**). Data are represented as mean ± standard deviation. **, *p* ≤ 0.01; ****, *p* ≤ 0.0001; ns, no statistical differences.

When the cardiovascular risk indexes were calculated, as shown in Figure 6, mice fed with the WD showed increased CRI I and II levels by 43.53% (58.56 ± 6.63, *p* < 0.0001) and 191.67% (0.35 ± 0.036, *p* < 0.0001) compared to the SD-fed mice (CRI I: 40.80 ± 0.81, CRI II: 0.12 ± 0.022). The MOMAST^®^ treatment counteracted this increase, reducing both CRI I by 25.94% (43.37 ± 3.83, *p* < 0.0001) and CRI II by 34.29% (0.23 ± 0.039, *p* < 0.0001), reaching similar values to the RYR-treated mice (CRI I: 42.21 ± 0.97, CRI II: 0.21 ± 0.031).

### 3.5. MOMAST^®^ Activates the SREBP-2/LDLR Pathway and Modulates the Active HMGCoAR Enzyme

**Figure 7:**
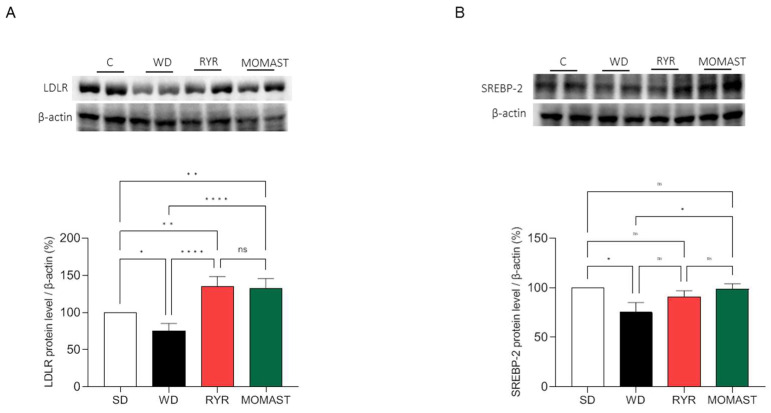
Modulation of the LDLR pathway by MOMAST^®^. MOMAST^®^ increases the LDLR (**A**) and SREBP-2 transcription factor (**B**) modulated by the Western diet. Data are represented as mean ± standard deviation. *, *p* ≤ 0.05); **, *p* ≤ 0.01; ****, *p* ≤ 0.0001; ns, no statistical difference.

SREBP-2 and LDLR proteins were quantified in the liver of the mice of the four experimental groups to investigate the effects of MOMAST^®^ on the LDLR pathway. Figure 5 shows that WD ingestion significantly reduced the LDLR protein level (Figure 7A) and the SREBP2 transcription factor (Figure 7B) by 24.78 ± 10.01% (*p* < 0.05) and 24.7 ± 9.95% (*p* < 0.05), respectively. The treatment with both RYR and MOMAST^®^ augmented LDLR by 34.7 ± 13.7% (*p* < 0.01) and 32.4 ± 13.39% (*p* < 0.01), respectively (Figure 7A), and in parallel, the SREBP-2 levels were increased, restoring the SD group values. In detail, the RYR and MOMAST^®^ diets lead to a decrease in SREBP-2 levels by 8.98 ± 6.08% and 1.13 ± 5.45%, respectively, versus the SD group Figure 5A.

**Figure 8:**
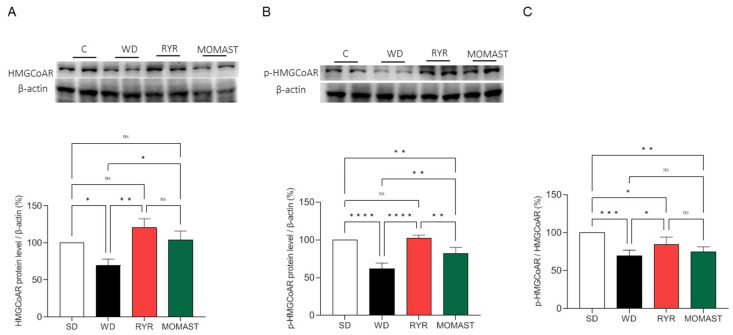
Effects of the MOMAST^®^ on HMGCoAR and p-HMGCoAR protein levels. Representative Western blot analyses of the HMGCoAR (**A**) and p-HMGCoAR (**B**) protein levels. The ratio of the inactive phosphorylated and active non-phosphorylated forms of HMGCoAR (**C**). Data are represented as mean ± standard deviation. *, *p* ≤ 0.05; **, *p* ≤ 0.01); ***, *p* ≤ 0.001; ****, *p* ≤ 0.0001; ns, no statistical differences.

The effects of MOMAST^®^ on the HMGCoAR protein levels were also evaluated by Western blotting. As shown in Figure 6, mice fed with the WD showed a decrease in HMGCoAR protein levels by 30.4 ± 11,91% compared to the SD-fed mice (Figure 8A). On the other hand, RYR treatment augmented HMGCoAR protein levels by 23.2 ± 25.78%, and MOMAST^®^ treatment by 3.8± 12.06% (Figure 8A). In parallel, in WD-fed mice, the SREBP-2 modulation led to a decline in the total phosphorylated and inactive p-HMGCoAR protein by 38.33 ± 7.79% (*p* < 0.0001, Figure 6B), while the RYR diets restored the p-HMGCoAR protein levels by up to 2.2 ± 4.23% and the MOMAST^®^ diet reduced these target levels by 17.84 ± 7.91%, versus the SD diet (*p* < 0.01; Figure 6B).

Furthermore, the p-HMGCoAR/HMGCoAR ratio was calculated. As shown in Figure 8C, the ratio was reduced by 30.47 ± 7.56 % (*p* < 0.001) in the WD-fed mice compared to the SD-fed mice. The treatment with both RYR and MOMAST^®^ reduced the p-HMGCoAR/HMGCoAR ratio by 15.35 ± 9.63 % (*p* < 0.05) and 25.17 ± 6.49 % (*p* < 0.01), respectively, compared to the SD group.

### 3.6. MOMAST^®^ Treatment Reduces the PCSK9 Protein Levels Increased by WD Ingestion

**Figure 9:**
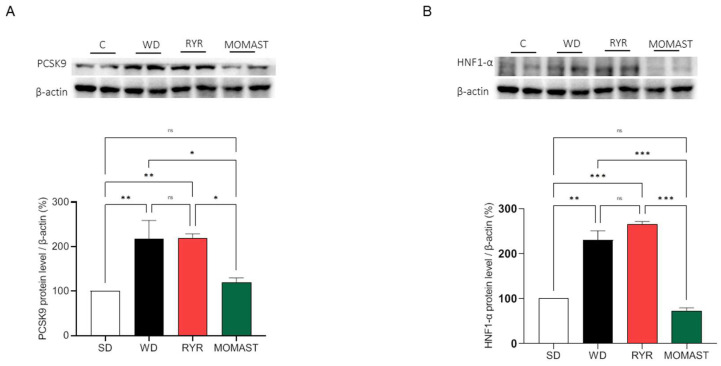
Effects of the MOMAST^®^ on PCSK9 and HNF1-α protein levels. The MOMAST^®^ counteracts the increase in PCSK9 (**A**) and its transcription factor HNF1-α (**B**) caused by the ingestion of WD. Data are represented as mean ± standard deviation. *, *p* ≤ 0.05; **, *p* ≤ 0.01; ***, *p* ≤ 0.001.

As indicated in Figure 7, the effects of MOMAST^®^ on the modulation of PCSK9 and its transcription factor HNF1-α were evaluated. Our results demonstrate that the ingestion of the WD leads to a significant augmentation of PCSK9 protein by 117.40 ± 41.59% (*p* < 0.01), whereas in RYR-fed mice the levels of this target increase up to 119.10 ± 9.72% (*p* < 0.01, Figure 9A). On the other hand, the mice treated with MOMAST^®^ showed a reduction in PCSK9 protein levels by up to 19.10 ± 10.72%, restoring the SD levels (Figure 9A).

This finding agrees with the HNF1-α transcription factor level modulations. Indeed, only MOMAST^®^ showed the capability to reduce the HNF1-α transcription factor levels. In detail, WD increased the HNF1-α protein levels by 130.0 ± 20.88% (*p* < 0.01), while RYR augmented the HNF1-α levels by 165.5 ± 5.87% (*p* < 0.001), and MOMAST^®^ diminished its levels by 27.68 ± 7.25%, compared with the SD group (Figure 9B).

## 4. Discussion

MOMAST^®^ is an innovative sustainable and bioactive ingredient rich in OH-Tyr and Tyr (Figure S1) which displays a potent direct antioxidant activity (Figure 2). The results indicated that MOMAST^®^ successfully scavenged the DPPH radicals and improved the FRAP capacity at 0.1 and 0.5 mg/mL, respectively.

This innovative ingredient showed the positive ability to modulate the cholesterol metabolism in human hepatic HepG2 cells with a mechanism of action which is not only linked to its antioxidant and anti-inflammatory behavior [4]. In order to obtain the *proof of concept* regarding its in vivo antioxidant and hypocholesterolemic effect, a suitable study was realized using mice in which the hypercholesterolemic condition was induced by feeding with the WD and then treating with MOMAST^®^ (20 mg/kg) and RYR (3% Monacolin) for eight weeks. The results observed in this study are independent of the daily food intake, which remained unchanged between the WD-fed groups. Firstly, the body weight results showed that MOMAST^®^ is capable of palliating the increase in body weight caused by WD ingestion (Figure 3). This is of great interest due to overweight and obesity having been pointed out as one of the pivotal risk factors in the development of the principal non-transmissible chronic diseases [11,12,13,14].

The findings clearly indicated that MOMAST^®^ improves the WD-induced oxidative stress measured in mice serum and liver (Figure 4). In more detail, it was observed that unlike in the RYR-fed mice, MOMAST^®^ showed an ability to decrease the DPPH radical compared with WD-fed mice, which showed an increase in DPPH radicals at the serum level. The in vivo antioxidant effect was also confirmed by performing a FRAP assay for mice serum. Indeed, MOMAST^®^-treated mice showed an increase in the FRAP levels (0.17 mmol equiv. Trolox/L) compared to the WD-fed ones (0.14 mmol equiv. Trolox/L, *p* = 0.039); also, in this case, RYR was not able to modulate the FRAP capacity in mice (Figure 4B). Furthermore, MOMAST^®^ showed an ability to reduce the hepatic WD-induced MDA, restoring the SD lipid peroxidation baseline levels, whereas RYR was confirmed to not modulate the lipid peroxidation in mice livers.

In addition, an increase in plasmatic lipid concentration was observed in the WD-fed mice compared to the SD-fed ones and a significant decrease in plasmatic lipid marker concentration in WD + MOMAST^®^-treated mice compared to the WD-fed mice, restoring the normal values observed in SD-fed mice, was successfully achieved. In particular, it was observed that MOMAST^®^ treatment reduces the WD-induced levels of TC and LDL and increases the HDL levels, without changing the TG levels (Figure 5A–D).

The behavior of MOMAST^®^ in the modulation of the plasmatic lipid profile is similar to WD + RYR^®^-treated mice; however, unlike it, in the WD + MOMAST^®^-treated mice a significantly augmentation of HDL was observed, suggesting a potentially better anti-atherogenic effect of MOMAST^®^ [15]. The Castelli risk index-I (TC/HDL ratio), also known as the cardiac risk ratio, reflects the formation of coronary plaques with a diagnostic value as good as the determination of total cholesterol [16,17]. On the other hand, the Castelli risk index-II (LDL/HDL ratio) has been shown to be an excellent predictor of cardiovascular risk [18]. Interestingly, MOMAST^®^, similarly to RYR, reduces both CRI-I and II compared to WD-fed mice, restoring the normal condition observed in the SD-fed mice, suggesting that MOMAST^®^ ameliorated the cardiovascular risk factors (Figure 6A,B). Using this hypercholesterolemic animal model, similarly to RYR behavior, we did not observe effects of MOMAST^®^ on triglyceride modulation (Figure 5B).

Basis on these results and to link the plasmatic lipid parameter modulation with the effects of MOMAST^®^ on the hepatic cholesterol metabolism pathway, Western blotting experiments were performed using liver homogenates. Our findings show that WD-fed mice presented reduced protein levels of LDLR, HMGCoAR, and SREBP-2 than SD-fed mice, and that the WD + MOMAST^®^-fed mice restored their protein levels of all the targets towards the normal condition observed in the SD-fed mice (Figure 7 and Figure 8A). HMGCoAR activity can be modulated by a reversible phosphorylation-dephosphorylation, with the phosphorylated form of the enzyme being inactive (70%) and the dephosphorylated form active (30%). Thus, when HMGCoAR is phosphorylated, the synthesis of de novo cholesterol is reduced [19,20]. Interestingly, in this study, it was observed that in the WD-fed mice the phosphorylated form of HMGCoAR was significantly reduced and that both RYR and MOMAST^®^ consumption increased the phosphorylated form of the enzyme towards the normal level observed in SD-fed mice (Figure 8B,C).

Overall, in agreement with the literature, the capability of MOMAST^®^ to modulate the LDLR-SREBP-2 pathway is similar to RYR treatment [21,22]. However, in line with a previous mechanistic study performed on HepG2 cells [4], it was confirmed that, unlike RYR, only MOMAST^®^ positively modulates the PCSK9 pathway via HNF1-α activation (Figure 7A,B). Notably, since the HNF1-α binding site is unique to the PCSK9 promoter and is not present in the LDLR promoter, the modulation of the PCSK9 transcription through HNF1-α does not affect the LDLR pathway. Thus, the co-regulation of PCSK9 by LDLR and other SREBP target genes is disconnected by the HNF1-α binding site [23]. In this context, it is important to highlight that a significant increase in HNF1-α and PCSK9 protein levels, respectively, were observed in WD-fed mice, which are clearly reduced by MOMAST^®^ toward the normal levels observed in SD-fed mice. On the contrary, the findings suggest that RYR is completely ineffective in the modulation of PCSK9 protein levels, being unable to reduce the increase in HNF1-α levels (Figure 9A,B).

## 5. Conclusions

In light of these results, MOMAST^®^ can be considered an innovative and sustainable antioxidant and hypocholesterolemic ingredient which is able to act with a mechanism of action which is different from RYR; therefore, it can be used alone or in combination with RYR (as strategy for reducing its amount of use) for the potential development of a new generation of dietary supplements or functional foods for the prevention of cardiovascular disease. In addition, further clinical studies can be performed to corroborate the ability of MOMAST to modulate cholesterol metabolism and oxidative stress in humans.

## 6. Patents

Patent n. 102021000019226 of the 20 July 2021 entitled ”*Processo produttivo di com-plessi polifenolici da acque di vegetazione olearie con processo fermentativo e relativi complessi polifenolici prodotti*”—inventors: Arnoldi A., Clodoveo M.L., Corbo F.F.R., Franchini C., Lammi C., Lentini, G. Lorenzo, V., Massari C.D, Milani G., Moretti P., and Pisano I.

## Data Availability

Not applicable.

## Supplementary Materials

The following supporting information can be downloaded at https://www.mdpi.com/article/10.3390/antiox12071335/s1, Figure S1: Chromatogram of the MOMAST^®^ phytocomplex.
