# Supplementary material for: MOMAST® Reduces the Plasmatic Lipid Profile and Oxidative Stress and Regulates Cholesterol Metabolism in a Hypercholesterolemic Mouse Model: The Proof of Concept of a Sustainable and Innovative Antioxidant and Hypocholesterolemic Ingredient
Source: Antioxidants (Basel). 2023 Jun 24;12(7):1335. doi: 10.3390/antiox12071335 (PMC10376191; doi:10.3390/antiox12071335)
Supplement: Supplementary file 1 [file antioxidants-12-01335-s001.zip › antioxidants-2448130-supplementary.pdf]

Supplementary Materials

MOMAST® reduces the plasmatic lipid profile and oxidative stress, and regulates the cholesterol metabolism in hypercholesterolemic mouse model: the *proof of concept* of a sustainable and innovative antioxidant and hypocholesterolemic ingredient

Ivan Cruz-Chamorro[1,2,†], Guillermo Santos-Sánchez[1,2,†], Eduardo Ponce-España[1,2], Carlotta Bollati[3], Lorenza d'Adduzio[3], Martina Bartolomei[3], Jianqiang Li[3], Antonio Carrillo-Vico[1,2], Carmen Lammi[3,*]

[1] Instituto de Biomedicina de Sevilla, IBiS/Hospital Universitario Virgen del Rocío/CSIC/Universidad de Sevilla, 41013, Seville, Spain
[2] Departamento de Bioquímica Médica y Biología Molecular e Inmunología, Facultad de Medicina, Universidad de Sevilla, 41009, Seville, Spain
[3] Department of Pharmaceutical Sciences, University of Milan, 20133 Milan, Italy
[†] These authors contributed equally.
[*] Correspondence: carmen.lammi@unimi.it; Tel.: +39-02-50319372

Citation: Cruz-Chamorro, I.; Santos-Sánchez, G.; Ponce-España, E.; Bollati, C.; d'Adduzio, L.; Bartolomei, M.; Li, J.; Carrillo-Vico, A.; Lammi, C. MOMAST® Reduces the Plasmatic Lipid Profile and Oxidative Stress, and Regulates Cholesterol Metabolism in a Hypercholesterolemic Mouse Model: The *Proof of Concept* of a Sustainable and Innovative Antioxidant and Hypocholesterolemic Ingredient. *Antioxidants* **2023**, *12*, 1335. https://doi.org/10.3390/antiox12071335

Academic Editor: Giovanni Caprioli

Received: 28 May 2023
Revised: 16 June 2023
Accepted: 21 June 2023
Published: 24 June 2023

Copyright: © 2023 by the authors. Submitted for possible open access publication under the terms and conditions of the Creative Commons Attribution (CC BY) license (https://creativecommons.org/licenses/by/4.0/).

2. Materials and Methods

2.1. Chemicals

Bovine serum albumin (BSA), β-mercaptoethanol, RIPA buffer, the antibody against phospho-HMGCoAR (Ser872) and the antibody against β-actin were bought from Sigma Aldrich (St. Louis, MO, USA). The antibody against HMGCoAR was bought from Abcam (Cambridge, UK). Lipid Peroxidation (MDA) Assay Kit (MAK085), phenylmethanesulfonyl fluoride (PMSF), Na-orthovanadate and the antibodies against rabbit Ig-horseradish peroxidase (HRP), mouse Ig-HRP, and SREBP-2 were purchased from Santa Cruz Biotechnology Inc. (Santa Cruz, CA, USA). The antibodies against the LDLR was bought from Pierce (Rockford, IL, USA). The antibodies against HNF1-α and PCSK9 were bought from GeneTex (Irvine, CA, USA). The inhibitor cocktail Complete Midi from Roche (Basel, Swiss). The chemiluminescent reagent was purchased from Euroclone (Milan, Italy). Mini protean TGX pre-cast gel 7.5% and Mini nitrocellulose Transfer Packs were purchased from BioRad (Hercules,CA, USA).

MOMAST® Description

Bioenutra S.R.L. (Italy) supplies the patented MOMAST® sample directly from the production process. MOMAST® sample is a phenolic complex, rich in tyrosol (4.2 g/kg, Tyr) and hydroxityrosol (12,4 g/kg, OH-Tyr). Its chromatogram is available as Supplementary Materials (Figure 1S).

2.2. in vitro MOMAST® antioxidant activity

2.2.1 2,2-diphenyl-1-picrylhydrazyl (DPPH) assay

To estimate the radical scavenging capacity of MOMAST®, the DPPH assay was carried out. Briefly, 45µL of DPPH solution (0.0125mM in methanol) was added to 15µL MOMAST® at 1-600 mg/mL. The scavenging reaction was performed in the dark for 30 min of incubation. After this time, the absorption was measured at 520 nm using a CLARIOstar Plus microplate reader (BMG Labtech, Ortenberg, Germany).

2.2.2 Ferric Reducing Antioxidant Power (FRAP) Assay

A volume of 15µL of a sample containing MOMAST® was combined with 140 µL of FRAP solution, which consisted of 0.83 mM TPTZ, 1.66 mM FeCl3× 6H2O, and 0.25 M acetate buffer(pH 3.6). The final concentrations of MOMAST® were 0.1 and 0.5 mg/mL. Following an incubation period of 30 minutes at 37 °C, the absorbance at 595 nm was measured usingaSynergyTMHT-multimode microplate reader (BiotekInstruments, Winooski,VT, USA). Themeasured values were extrapolated using a Trolox (Sigma-Aldrich) standard curve.

2.3. Study design

26 C57BL/6 4-weeks-old mouse were housed at the Animal Facility of the Instituto de Biomedicina de Sevilla (IBiS) under standard conditions and were fed with WD (45% energy from fat, TestDiet, St. Louis, MO, USA, n = 21) or with standard diet (SD, Rodent maintenance diet, ENVIGO, IN, USA, n = 5). When mice were 6-weeks-old, they were separated in three group and intragastrically treated with: **1**) saline (control group, C, n = 7), **2**) red yeast rice (3% Monacolin K, RYR, ADVA s.r.l., Italy, n = 7), or **3**) MOMAST® (Bioenutra s.r.l., Italy, n = 7) at 20 mg/kg for eight weeks.

The individual body weight and the daily food intake were weekly recorded. At the final of the experiment, an intraperitoneal injection of sodium thiopental (B. Braun Medica SA, Barcelona, Spain) was used to sacrifice the 12 h fasted-mice. By cardiac puncture the blood was collected in the MiniCollect tubes (Greiner Bio-One, Kremsmünster, Austria), which was centrifuged at 3,000 g for 10 minutes at 4 °C to obtain the serum that was immediately frozen and stored at −20 °C until use. Also, the liver was extracted, weighed, and finally rapidly frozen and stored at −80 °C until use.

All procedures were approved by the Ethical Committee (reference number 16/03/2023/004).

2.4. Biochemical parameters

All biochemical parameters were quantified in the serum samples using the COBAS E601 modular analyzer (Roche Diagnostic, Bassel, Switzerland). Castelli risk index (CRI) I and II were calculated as TC/HDL and LDL/HDL, respectively.

2.5. In vivo antioxidant activity of MOMAST®

2.5.1 DPPH and FRAP assays

Serum antioxidant capacity was evaluated using DPPH and FRAP assays. Briefly, for the FRAP assay,15 µL of serum was combined with 140 µL of FRAP solution, while for DPPH assay,15µL of serum was added to 45µL of DPPH solution. Both assays test were carried out according to the manufacture’s instruction.

2.5.2. Determination of liver malondialdehyde (MDA) levels

Each liver sample (10 mg) was homogenized in 300 µL ice-cold MDA lysis buffer containing 3 µL of BHT (100×) and was centrifuged at 13,000× g for 10 min. To form the MDA- thiobarbituric acid (TBA) adduct, 600 µL of the TBA solution were added into each vial containing 200 µL of samples and incubated at 95 ◦C for 60 min, then cooled to RT for 10 min in an ice bath. For analysis, 100 µL of each reaction mixture were pipetted into a 96 well plate and the absorbance was measured at 532 nm using the Synergy H1 fluorescence plate reader (Biotek, Bad Friedrichshall, Germany).

2.6. Western-blot analysis

Tissues were homogenized in a lysis buffer (RIPA buffer +protease inhibitor cocktail + 1:100 PMSF + 1:100 Na-orthovanadate + 1:1000 β-mercaptoethanol). Protein concentration was determined by the Bradford's method and 50 µg of total proteins were run into a precast 7.5% sodium dodecyl sulfate-polyacrylamide gel (SDS-PAGE) at 130 V for 45 min and then transferred to a nitrocellulose membrane (Mini nitrocellulose Transfer Packs, BioRad, Hercules, CA, USA) using a Trans-Blot Turbo (BioRad) at 1.3 A, 25 V for 7 min, and stained with Ponceau red solution (Sigma-Aldrich). To investigate proteins of different sizes on the same membrane, the membrane was cut into horizontal stripes. The milk or BSA blocked membrane were overnight incubated at 4 °C with primary antibodies against SREBP2, HMGCoAR, LDLR, phospho HMGCoAR (Ser872), PCSK9, HNF1-α, and β-actin. After washing, the blots were incubated with secondary antibodies conjugated with horseradish peroxidase. A chemiluminescent reagent (Euroclone, Milan, Italy) was used to visualize target proteins and the densitometry analysis was performed using the software Image Lab 6.1 (BioRad). The internal control β-actin was used to normalize loading variations.

2.7. Statistical analysis

All the data sets were checked for normal distribution by D'Agostino and Pearson test. Since they are all normally distributed with p-values < 0.05, statistical analysis was carried out by One-way ANOVA followed by Tukey's post-hoc analysis (GraphPad Software 9, San Diego, CA, USA). Values were expressed as means ± standard deviation; p-values ≤ 0.05 were considered to be significant.

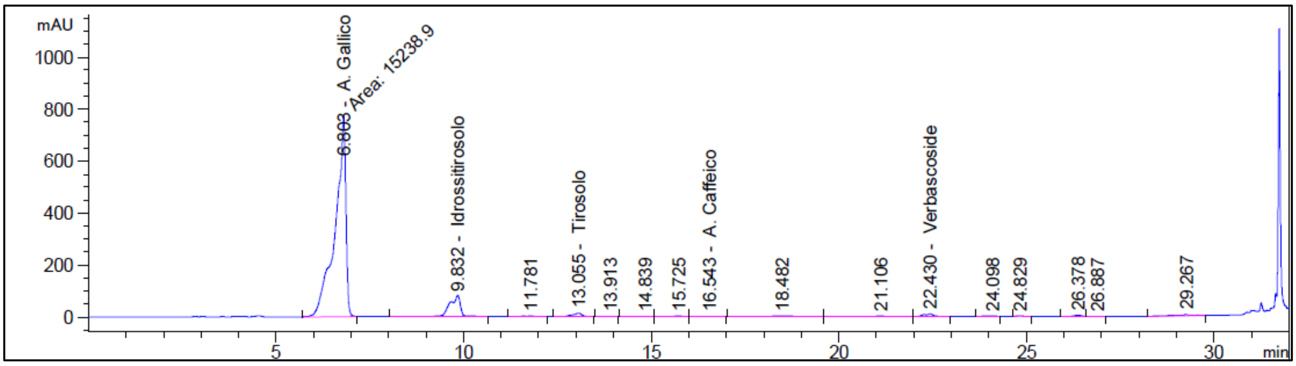

Figure S1: Chromatogram of the MOMAST® phytocomplex.
